# Mycobiome in the Gut: A Multiperspective Review

**DOI:** 10.1155/2020/9560684

**Published:** 2020-04-04

**Authors:** Voon Kin Chin, Voon Chen Yong, Pei Pei Chong, Syafinaz Amin Nordin, Rusliza Basir, Maha Abdullah

**Affiliations:** ^1^School of Biosciences, Faculty of Health and Medical Sciences, Taylor's University Lakeside Campus, 47500 Subang Jaya, Malaysia; ^2^Department of Medical Microbiology and Parasitology, Faculty of Medicine and Health Sciences, Universiti Putra Malaysia, 43400 Serdang, Selangor, Malaysia; ^3^Department of Human Anatomy, Faculty of Medicine and Health Sciences, Universiti Putra Malaysia, 43400 Serdang, Selangor, Malaysia; ^4^Department of Pathology, Faculty of Medicine and Health Sciences, Universiti Putra Malaysia, 43400 Serdang, Selangor, Malaysia

## Abstract

Human gut is home to a diverse and complex microbial ecosystem encompassing bacteria, viruses, parasites, fungi, and other microorganisms that have an undisputable role in maintaining good health for the host. Studies on the interplay between microbiota in the gut and various human diseases remain the key focus among many researchers. Nevertheless, advances in sequencing technologies and computational biology have helped us to identify a diversity of fungal community that reside in the gut known as the mycobiome. Although studies on gut mycobiome are still in its infancy, numerous sources have reported its potential role in host homeostasis and disease development. Nonetheless, the actual mechanism of its involvement remains largely unknown and underexplored. Thus, in this review, we attempt to discuss the recent advances in gut mycobiome research from multiple perspectives. This includes understanding the composition of fungal communities in the gut and the involvement of gut mycobiome in host immunity and gut-brain axis. Further, we also discuss on multibiome interactions in the gut with emphasis on fungi-bacteria interaction and the influence of diet in shaping gut mycobiome composition. This review also highlights the relation between fungal metabolites and gut mycobiota in human homeostasis and the role of gut mycobiome in various human diseases. This multiperspective review on gut mycobiome could perhaps shed new light for future studies in the mycobiome research area.

## 1. Introduction

Human gut is a complex ecosystem inhabited by a myriad of microorganisms including bacteria, fungi, archae, and viruses [1]. Thus far, studies on gut bacteria or gut “microbiome” have received the most attention due to the abundance of bacterial flora present in the gut. For example, large-scale projects including Metagenomics of the Human Intestinal Tract (MetaHIT) and National Institutes of Health (NIH) Human Microbiome Project (HMP) were among the projects that were initiated to study the composition of bacterial flora and their impacts on human health [1, 2]. Nevertheless, the shift in focus towards the “rare biosphere” [3] in the gut, particularly on fungi, has gathered increasing traction nowadays. Studies on “mycobiome,” a term used to describe the fungal community of the microbiome, have escalated despite still being in its infancy stage as it may be significant in the context of human health and diseases [4].

The studies on human gut mycobiome have received little attention for the past decades because fungal presence is relatively insignificant in the gut compared to the bacterial communities. In addition to that, fungi have been traditionally studied by culture-dependent methods [5, 6], which limits the in-depth understanding of the fungal microbiota. Nonetheless, recent advances in deep-sequencing technologies and bioinformatics analysis have shed light on the complexity of the fungal communities that reside on both mucosal and luminal surfaces in the gut and further highlighted our current understanding on this poorly understood compartment that reside in the gut. Increasing evidences have demonstrated the undisputable role of fungal components in driving the pathogenesis of various gut-associated and metabolic diseases [7–9]. In addition to that, specific fungi possess the ability to modulate host immune response and could be a risk factor for immunological disorders seen in genetically susceptible individuals [10]. More profoundly, gut mycobiome could be the reservoir for opportunistic pathogens in immunocompromised hosts [11–13]. These clearly indicate that gut mycobiome is crucial in host homeostasis and disease development. Thus, in this review, we seek to summarize the role of gut mycobiota from multiple perspectives and to highlight the latest research on gut mycobiome on disease development, in order to provide new insights and productive direction for future studies in this newly emerged research area.

## 2. Fungal Communities in Gut

In general, fungi constitute a minor component of the entire gut microbiome. Recent shotgun metagenomics sequencing analysis has revealed that fungi consist of nearly 0.1% of the total microbes in the gut [1, 14]. On the other hand, despite numerous published data on gut mycobiome, the fungal communities in the gut remain poorly understood. There is no consensus in defining a healthy gut mycobiome due to a variety of factors such as low abundance and diversity of fungi in the gut, temporal instability of gut mycobiota throughout the development periods, and high intervolunteer and intravolunteer variability of the gut mycobiome [15, 16]. Nevertheless, the fungal communities in the gut have been highlighted in many studies [8, 16, 17]. In terms of phyla, thus far, most studies have suggested that Ascomycota is the most predominant phylum found in the gut, followed by Zygomycota and Basidiomycota phyla [15, 18–20]. Meanwhile, in the context of genus, a recent review by Hallen-Adams and coworkers had identified potential fungal species that inhabit the intestinal niche, belonging to the genera *Candida*, *Cryptococcus*, *Malassezia*, *Aspergillus*, *Saccharomyces*, *Galactomyces*, *Trichosporon*, and *Cladosporium* [21]. Recently, through the Human Microbiome Project (HMP), Nash and coworkers (2017) sequenced 317 stool samples in a healthy cohort via Internal Transcribed Spacer 2 (ITS2) region and 18S rRNA gene. The authors reported that gut mycobiota is mainly dominated by *Malassezia*, *Candida*, and *Saccharomyces*, with *S. cerevisiae*, *M. restricta*, and *C. albicans* identified in 96.8%, 88.3%, and 80.8% of the samples, respectively [16]. Reports from earlier studies using culture-dependent analysis showed that less than 30% of fungal species are present in the human gut [22, 23]. Collectively, these studies suggest that gut mycobiome is relatively low in diversity as compared to bacterial communities, and the high prevalence of several fungal species identified across samples further suggested that a core mycobiota may exist in the gut.

In the context of fungal development in infants, Schloss and coworkers performed analysis on gut microbiome profile between family members and unrelated subjects and discovered that similar gut microbiome profile are identified in people sharing same life history and environment [24]. A study by Palmer et al. demonstrated that variable levels of fungi were detected from the stool samples of healthy full-term infants after birth and also at defined intervals throughout the first year of life using microarray and PCR analysis. Also, the authors reported the absence of fungal species from the initial stool sample in one of the healthy infants, where the mother of the infant showed detectable fungal levels in the vagina, raising the possibility that vertical transmission (vaginal microbiota) has minimal impact on shaping the fungal colonization in the infant [25]. Bliss et al. had demonstrated vertical and horizontal transmission of *C. albicans* from mother to infant through DNA fingerprinting techniques [26]. On the other hand, diet could affect the composition of the infant's mycobiota as well. For instance, *Candida* species have been identified in breast-feeding women with symptoms of mammary candidiasis [27]. A study by LaTuga et al. identified several fungal species in extremely low birth weight infants in their first postnatal month. These fungal species include *S. cerevisiae* followed by *Candida* spp., *Cladosporium* spp., and *Cryptococcus* spp. [28]. Another study also reported a lack of fungal diversity and richness in eleven infants using combined NGS technology (targeted on fungal ITS2 amplicons) with qPCR analysis. The most predominant fungi identified in this study were *C. albicans*, *C. parapsilosis*, and *Leptosphaerulina*, with *C. albicans* being detected in all infants. The authors speculate that the relatively low diversity of fungi in infants as compared to adults could be due to age factor where these infants may not yet been fully colonized by a myriad of different fungal species. Further, the authors highlighted the challenge of detecting low abundance fungi, where low levels of *Candida parapsilosis* and *Candida krusei* sequences were detected in a few samples; however, their presence was only detected in one sample via species-specific qPCR [29]. On the other hand, a study by Strati et al. demonstrated the influence of age and gender on the gut fungal composition. In this study, the authors reported that infants and children have higher fungal richness than in adults via culture-independent analysis. Meanwhile, the diversity and fungal richness was also higher in female than male healthy subjects [30]. Together, the diversity of fungal composition, the timing and the mechanism involved in assembling fungi into gut microbiome, the influence of genetic and environmental factors in shaping the infant mycobiota, and the contribution of fungi to the microbiome phenotype remain to be explored further.

## 3. Gut Mycobiome and Host Interactions

**Figure 1:**
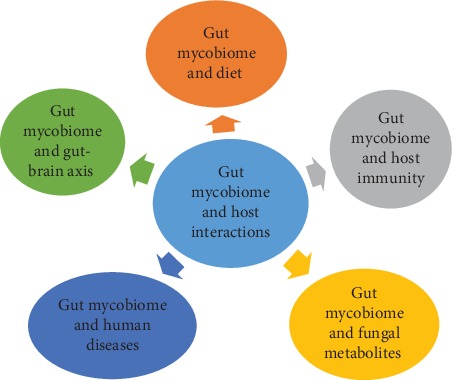
The interactions between gut mycobiome and host in various areas.

As discussed previously, gut ecosystem remains a complex environment harboured by various “microscopic” and “macroscopic” organisms. It is believed that the ecosystem in the gut is maintained via multiple interactions among gut members (bacteria, fungi, and virus) as suggested by Filyk and Osborne [31]. On the other hand, accumulated evidences also suggest significant involvement of gut fungi in maintaining host dynamic and the potential of utilizing fungal metabolites in clinical applications. Thus, in this section, we will discuss interaction between fungi with host, deliberate on fungal-bacteria interaction and gut-brain axis, examine the impact of gut fungi on diet and host immunity, and also highlight the influence of gut fungi on health and diseases (Figure 1).

### 3.1. Fungal-Bacteria Interactions

Generally, the dialogue between fungi and bacteria is studied through the induction of dysbiosis in the gut followed by treatment with either antifungal or antibacterial drugs. It is well known that antibiotics specific to anaerobic bacteria or broad-spectrum antibiotics can have differential impacts on fungal susceptibility, in particular *C. albicans* [32, 33]. Some studies even suggested that the administration of *C. albicans* to mice after antibiotic exposure could significantly change the gut microbiome composition, from phyla to family level, and the changes are irreversible in the long run [34, 35]. Further, mycobiome equilibrium is known to have impact on microbiome stability. Such impact is demonstrated in mice model of dextran sulphate sodium- (DSS-) induced colitis. In this model, it was found that the prescription of antifungal drug in the mice had significantly reduced the fungal diversity along with the increase of pathogenic bacterial diversity, which in turn exacerbated the severity of colitis inflammation [36]. On the other hand, a study by Jiang et al. demonstrated that commensal fungi such as *C. albicans* or *S. cerevisiae* can functionally replace intestinal bacteria in the event of bacterial dysbiosis after antibiotic exposition. Moreover, these fungal species confer protection against colitis and influenza A virus infection through alleviation of mucosal tissue injuries and host immune modulation [37]. Another robust example of fungal-bacteria interaction is secretion of extracellular enzymes such as phosphatases and proteases by *S. boulardii*, which aids in deactivating the toxins produced by *C. difficile* and *E. coli* [38, 39]. Furthermore, studies had shown that *R. gnavus* and *C. albicans* can cause lesion formation in the gut by degrading the protective mucin layer through the action of mucolytic enzymes [40, 41]. Meanwhile, some fatty acid metabolites secreted by bacterial flora also seem to modulate *C. albicans* germination [42]. A more recent study by García et al. demonstrated that gut microbial metabolites inhibit the invasion of human enterocytes and *C. albicans* hyphal growth through the target of rapamycin (TOR) signaling pathway [43]. Other than that, interaction between fungal and bacterial cells can cause adverse effects on host via proinflammatory cytokines secretion, which result in apoptotic cell death and oxidative damage on host [44].

Fungi and bacterial cells are also believed to interact with each other within a biofilm habitat, a so called “mixed species biofilm.” Such a habitat can help them to persistently colonize and survive in specific microenvironments such as gut, skin, and oral cavity. Additionally, this “mixed species biofilm” can give extra protection against antimicrobial agents and host immune evasion [45]. Notably, this “mixed species biofilm” provides mutual benefits for both bacterial and fungal cells. Fungi can strengthen their virulence determinants within the biofilm habitat, such as increasing the ability to invade host through hyphal induction and production of extracellular enzymes such as aspartic proteinases. Meanwhile, bacteria may benefit from this habitat via the increase in their resistance towards antimicrobial treatment [45]. Kalan et al. had demonstrated a rapid formation of mixed species biofilms between *Trichosporon asahii* and *Staphylococcus simulans* or *C. albicans* and *Citrobacter freundii in vitro*, revealing a close relation between fungal and bacterial cells [46]. Similarly, Hoarau et al. reported formation of robust biofilm between *C. tropicalis*, *S. marcescens*, and *E. coli* in *in vitro* biofilm model [47]. Other studies also demonstrated that production of lipopolysaccharides *by S. marcescens* and *E. coli* enhanced fungal biofilm maturation [48, 49]. Clearly, there is a strong connection between fungal and bacteria cells. A more in-depth analysis of this interkingdom interaction can provide us with more information on the gut-associated pathogenesis driven by this interaction.

### 3.2. Mycobiome and Gut-Brain Axis

It is interesting that besides gut microbiome, fungi are involved in the gut-brain axis (GBA). Growing evidences from both clinical and experimental studies suggest that fungi are involved in the bidirectional communication between brain and gut through neuro-immuno-endocrine mediators, which is comparable to microbiome-gut-brain axis [50]. An excellent example of this mycobiome-gut-brain communication is via a study by Botschuijver et al., where the authors investigated the association of intestinal fungal components in patients with inflammatory bowel syndrome (IBS) and in a rat model of visceral hypersensitivity. In the study, the authors observed a mycobiome dysbiosis in the rat model of visceral hypersensitivity, where administration of fungicide, cecal mycobiomes, and soluble *β*-glucans improved hypersensitized rats [51].

### 3.3. Gut Mycobiome and Immunity

The crosstalk between fungi and host immunity had been extensively reviewed [7, 44]. Of all, Dectin-1 appears as one of the most crucial pattern recognition receptors (PRRs) in shaping fungal immunity [10, 52]. Dectin-1 interacts with *β*-1,3 glucan motif found on the fungal cell walls and elicits host immune response against them. The importance of Dectin-1 has been implicated in the mouse model of DSS-induced colitis where Dectin-1 knockout mice experienced more severe colitis when compared to wild-type mice. In addition, expansion in *Candida* and *Trichosporon* genera and a decrease in *Saccharomyces* genus, together with increased of proinflammatory cytokines, IFN-*γ*, IL-17, and TNF-*α*, also aggravated inflammation in Dectin-1 knockout mice [10]. Further, clinical study has pointed out that polymorphisms in Dectin-1 gene are likely to contribute towards disease exacerbation in patients with ulcerative colitis (UC) [10]. In addition, other evidences also showed that deficiency in Dectin-1 is also linked with increased gastrointestinal colonization with *Candida* species in patients who received transplants [53, 54]. Taken together, these studies provide evidence on the protective role Dectin-1 in fungal infection and the importance of Dectin-1 to keep fungal in check.

Caspase recruitment domain-containing protein 9 (CARD9), a crucial downstream molecule for antifungal receptors including C-lectin receptors, is also implicated in defense against fungi. In the mice model of colitis, CARD9 knockout mice had increased antifungal antibodies, and the severity of colitis was mitigated upon antifungal treatment, suggesting the protective role of CARD9 signaling against fungi [55]. Meanwhile, IL-17, an effector cytokine for Th-17 helper cells, is involved in the mucosal immune response against fungi. The roles of IL-17 during mucosal fungal infections have been documented in both clinical and animal experimental studies [56–58]. Nevertheless, it remains obscure on the effect of IL-17 on gut mycobiota. One clinical study has reported that higher incidence of fungal infections accompanied with severe intestinal pathology were observed in patients with Crohn's disease upon IL-17A blockade, indicating the possible role of IL-17 pathway in regulating fungal communities in the gut [59]. A review by Conti et al. also suggested the pertinent role of IL-17 against opportunistic *C. albicans* [56]. On the other hand, IL-22, a cytokine that is similar to IL-17, is also closely linked with mucosal immunity against fungi. It has been demonstrated that IL-22 regulates gastrointestinal fungi, where mice lacking IL-22 are more prone to gastrointestinal candidiasis upon intragastric challenge with *C. albicans* [60]. Furthermore, both IL-17 and IL-22 are potent inducers of antimicrobial peptides (AMPs) by epithelial cells, which have indisputable role in clearing mucosal *Candida* spp. and *Aspergillus* spp. infections [61–64].

Crosstalk between gut mycobiome and host immune system can modulate the disease outcome. For example, Qamar et al. reported protective effect of *Saccharomyces boulardii* against *Clostridium difficile* colitis-induced mice model. In this study, the authors showed that administration of *S. boulardii* stimulates production of intestinal immunoglobulin A (IgA) against Clostridium difficile toxin A in mice [65]. In another study, Thomas and coworkers reported the anti-inflammatory effects of *S. boulardii* in patients with inflammatory bowel diseases, mainly via inhibition of T and dendritic cells activation, reduced levels of proinflammatory cytokines including tumor necrosis factor-*α* and interleukin- (IL-) 6 and increase the production of IL-10, which consequently promotes epithelial restitution relevant in IBD [66]. On the other hand, crosstalk between immune system and fungi may influence bacteria in the gut and vice versa. A study by Tang et al. demonstrated that the colon of Clec7a−/− mice was protected by *Lactobacillus murinus*-induced regulatory T cell expansion in the absence of *Candida* species. Nevertheless, the presence of *C. tropicalis* seems to cancel this protective effect and exacerbates intestinal inflammation [67]. Meanwhile, colonization of *Candida* species as seen in Card9−/− mice also reduced the populations of tryptophan-metabolizing bacteria, including *lactobacilli*, which intensify the severity of colitis in Card9−/− mice. The reduction of lactobacilli is accompanied with reduced levels of aryl hydrocarbon receptor (AHR) ligands, Reg3g, Reg3b, and Il22 expression in the colons of Card9−/− mice [68]. In addition to that, fungal microbiota also helped in shaping the host immune system in regards to the development of colorectal cancer. A recent study by Wang et al. demonstrated the adaptor protein CARD9 confers protection against colon cancer through restriction of Mycobiota-Mediated Expansion of Myeloid-Derived Suppressor Cells (MDSCs) in mice model [69].

Fungal species such as *S. cerevisiae* and *C. albicans* are capable of modifying immune response in a significant way. For example, chitin from *S. cerevisiae* is able to induce “trained immunity (a de facto immune memory of the innate immunity)” in monocytes in a strain dependent manner by increasing cytokine productions such as TNF-*α* and IL-6 and through direct antimicrobial activity upon stimulation with bacterial, fungal, and TLR ligands [70]. Similarly, *C. albicans* can induce “trained immunity” along with functional reprogramming of monocytes, which confers protection against reinfection [71]. Taken together, these studies suggest that “trained immunity” is important in maintaining gut immune homeostasis and confer protection on host against invading pathogens.

During gut-brain communication, the effect of intestinal inflammation on the central nervous system is often associated with anxiety, depression, or “sickness behavior” [72]. These psychological behavioral changes are always considered as comorbidities in patients with persistent intestinal inflammation, including inflammatory bowel diseases and irritable bowel diseases [73]. The underlying mechanism could be due to imbalances in serotonergic activity and hyperreactivity of hypothalamic-pituitary-adrenal axis (HPPA) [74, 75]. It is believed that this systemic effect is mediated by host immune factors, especially cytokines including interleukin-6 (IL-6), interleukin-1*β* (IL-1*β*), and tumor necrosis factor (TNF) [76]. Studies have shown that fungi such as *C. albicans*, *S. cerevisiae*, and *A. fumigatus* are able to modulate cytokine expression, particularly IL-6 [70, 77]. Thus, it is speculated that gut mycobiota may elicit local immune response at the gut site, where these immune mediators, especially cytokines will cross the blood-brain barrier (BBB), and reaching the brain to stimulate specific brain areas, in particular the hypothalamus and circumventricular organs [78].

Meanwhile, there is increasing evidences showing the influence of gut mycobiota on extragastrointestinal organs immune responses. A typical example is through a study by Wheeler et al., where the authors demonstrated an aggravation of allergic airway disease accompanied with altered gut mycobiota prior to fluconazole administration in mice with DSS-induced colitis. In this study, an expansion of *Wallemia sebi*, *Aspergillus amstelodami*, and *Epicoccum nigrum* and a reduction of *Penicillium brevicompactum* and *C. tropicalis* were observed [79]. Meanwhile, McAleer et al. demonstrated the ability of gut microbiota in shaping the pulmonary immune response upon *A. fumigatus* oropharyngeal challenge. In this study, mice treated with vancomycin for a month had reduced levels of IL-17 and IL-22 and increased the level of IL-4 in the lungs, which indicates the involvement of Th-17 and Th-2 responses against fungi in the lungs [80]. A recent study by Skalski et al. demonstrated that an expansion of *Wallemia* spp. (*W. mellicola*) in the gut modified the pulmonary immune response and intensified the airway inflammation in the intratracheal house dust mite (HDM) mice model of allergic airways disease. Additionally, *Wallemia* was not detected in the lung, suggesting the ability of gastrointestinal colonization of *Wallemia* to modulate lung immune response remotely. Further analysis of the gut mycobiota revealed that the expansion of *W. mellicola* in the gut was associated with perturbation in both fungal and bacteria communities [81]. Another study by Li et al. reported that gut fungal dysbiosis induced by fluconazole persistently exacerbates allergic airway disease (AAD) in mice, where this effect is mainly via fungal sensing by gut-resident CX3CR1^+^ MNPs on peripheral immunity [82]. Overall, these studies revealed a clear link between gut mycobiota, pulmonary immune responses, and lung diseases, which further consolidate the idea of the existence of mycobiota-gut-lung axis.

### 3.4. Gut Mycobiome and Diet

Diet could be one of the determinants in driving the changes in gut fungal mycobiota composition between individuals. An investigation on the link between diet and fungal mycobiota has been carried out by Hoffmann and his coworkers using culture-independent analysis. In the study, the authors had identified 66 fungal genera, with *Candida*, *Cladosporium*, and *Saccharomyces* being the most common genera identified. The authors speculate that the high prevalence of *Saccharomyces* could be due to consumption of yeast-containing foods such as beer and bread while high level of *Candida* was strongly correlated with the recent consumption of carbohydrates. Although this study has identified various fungal communities in the gut, one of the concerns raised in this study is whether these fungal mycobiota identified are permanent residents in the gut or just transient species [19]. Meanwhile, another study had profiled the short-term effect of plant and animal diet on the gut microbiome. In this study, David et al. showed that short-term diet consisting of both animal and plant products alters the microbial community structure. In addition to that, diversified fungal genera including *Scopulariopsis*, *Penicillium*, *Debaryomyces*, and *Candida* were identified in this study [83]. Moreover, diet therapy and antibiotics seem to reduce the abundance of fungal species in patients with Crohn's disease, as implicated by Lewis et al. [84]. Collectively, studies on the impact of diet on gut mycobiome are still in its infancy. Nonetheless, more studies are warranted to evaluate and to associate the effect of diet and food-associated fungi in shaping the diversity, composition, and functionality of human microbiome as well as the effect on diet on gut-associated diseases.

### 3.5. Gut Mycobiome and Fungal Metabolites

Since the invention of first antibiotic-Penicillin G from *Penicillium notatum* by Sir Alexander Fleming in 1928, the era of using fungi for medicinal purposes has started. Fungi have shown indispensable role in various areas, including food, biotechnology, and pharmaceutical industries. Fungi also possess great potential in producing a broad spectrum of metabolites, which can be applied for medication or therapeutic purposes. For example, griseofulvin isolated from *Penicillium griseofulvum* served as antimycotic drug [85], while fusidic acid from *Fusidium coccineum* [86] and cephalosporins from *Acremonium chrysogenum* act as antibacterial agents, and lovastatin isolated from *Aspergillus terreus* and mevastatin from *Penicillium citrinum* [87] can be used as lipid lowering agents.

Besides this, similar like bacteria, fungi can express metabolites which can influence host homeostasis and exert biological effects on host, as part of fungi-host interactions [17]. Fungal species such as *Saccharomyces boulardii*, *C. albicans*, and *Saccharomyces cerevisiae* may secrete molecules like farnesol, fusel alcohols, tyrosol, and fatty acids, which are autoregulatory molecules of growth. These molecules enable fungal cells to regulate adhesion, yeast-to-hyphae transition and biofilm formation themselves, which in turn facilitate the colonization, invasion, and dissemination in host [88]. On the other hand, polysacharide *β*1, 3-glucan is a fungal-derived molecule found in the inner cell wall of *C. albicans*. *β*1, 3-glucan has strong link with host immunity. A recent study suggested that *β*1, 3-glucan can trigger “trained immunity” prior to exposure to monocytes, generating more robust immune response upon fungal reinfection. Additionally, *β*1, 3-glucan can induce epigenetic changes including histone methylation upon exposure to monocytes [71].

Meanwhile, the probiotic yeast *Saccharomyces boulardii* is able to produce a low molecular weight, water soluble anti-inflammatory factor, which is capable to mediate signal transduction pathway in host cells, such as NF-*κ*B. It also helps to conserve the tight junction integrity between enterocytes in the small intestine and modulates signal transduction pathway during enteropathogenic *E. coli* infection [89, 90]. Other than that, *Saccharomyces boulardii* has been applied in treating various gastrointestinal disorders, as reviewed by Kelesidis. Nonetheless, safety issues concerned with using fungi as probiotics need to be considered, especially in immunocompromised or critically ill patients. These concerns include the possibility of developing fungemia after fungi treatment, gastrointestinal allergic reaction, and the environment risk prior to exposure of fungi in the air [91]. *Saccharomyces boulardii* may also exert trophic effect on the intestinal enterocytes via endoluminal release of polyamines. A study by Buts et al. reported that daily administration of lyophilized *S. boulardii* had significantly increased sucrase and maltase activities in rats' intestines [92]. The actual mechanism remains unknown, but it is likely through the release of spermine and spermidine. Meanwhile, *S. boulardii* produces a 54 kDa serine protease which can directly inhibit *Clostridium difficile* toxin A in rat ileum and *Clostridium difficile* toxins A and B in human colonic mucosa [38, 93]. Additionally, it also produces a 63-kDa phosphatase, which degrades *Escherichia coli* endotoxin by dephosphorylation [39]. Another study also documented the effect of Capric acid produced by *S. boulardii* in inducing adhesion, yeast-to-hyphae transition, and biofilm formation [94]. Moreover, administration of *S. boulardii* increases short-chain fatty acids (SCFAs), particularly butyrate, which may confer protective effect in total enteral nutrition- (TEN-) induced diarrhea [95]. Similarly, SCFAs produced by *Malassezia* species through enzymatic reaction (lipases and phospholipases) on host triglycerides found abundantly on skin could serve as metabolic sources for fungi [96, 97].

Some of the fungal-derived molecules are also known to mediate the interaction between fungal and bacteria. For example, ethanol from *Saccharomyces cerevisiae* is able to trigger the growth of Acinetobacter (*A. baumannii*, *A. johnsonii*, *A. haemolyticus*, and *A. radioresistens*) *in vitro* [98]. Farnesol, a small molecule produced by *C. albicans*, can also modify quorum sensing regulation in *Pseudomonas aeruginosa* [99, 100]. This showed that fungi are able to produce assorted secondary metabolites, and more studies can be conducted to shed light on the contribution of fungal metabolites produced in the gut on health and disease.

### 3.6. Gut Mycobiome and Disease Susceptibility

The significant involvement of fungi in the development and progression of human diseases have been reviewed extensively recently. Often, fungal dysbiosis contributes towards development and progression of several human diseases. A summary of the significant involvement of fungi in human diseases is depicted in Table 1.

#### 3.6.1. Inflammatory Bowel Disease (IBD)

Inflammatory bowel disease (IBD) is a group of intestinal diseases characterized by persistent inflammation of the digestive tract. The two most common diseases under IBD include Crohn's disease (CD) and ulcerative colitis (UC). A number of sources have proven a causal relation between gut mycobiome and IBD. For example, Ott et al. highlighted the differences between IBD with mucosal and fecal microbiota. The authors surmised that there is an alteration in the diversity and composition of fungal microbiota between patients with IBD and controls. However, the authors reported that there are no significant changes between CD and UC [101]. Subsequently, another study by Li et al. demonstrated that fungal dysbiosis in CD are associated with mucosal inflammation in patients with CD. In this study, the authors reported that the feces of patients with CD were characterized by the abundance of *Aspergillus clavatus*, *C. neoformans*, and *Candida albicans*, while *Alternaria brassicicola*, *Gibberella moniliformis*, *Cryptococcus neoformans*, and *Candida* spp. were reported in the inflamed mucosa [102]. Additionally, the diversity and richness of fungal species identified in this study were associated with the expression of, IFN-*γ*, IL-10, or TNF-*α*. Meanwhile, a study by Lewis et al. had identified fungal dysbiosis in patients with CD with an increase in *Saccharomyces cerevisiae*, *Cyberlindnera jadinii*, *Clavispora lusitaniae*, *Kluyveromyces marxianus*, and *Candida albicans*, accompanied with a change in bacteria composition [84]. A study by Hoarau et al. reported that an expansion of *C. tropicalis* is found in patients with Crohn's disease as compared to their healthy relatives. Furthermore, the authors reported a positive correlation between *C. tropicalis*, *S. marcescens*, and *E. coli*, suggesting that an interkingdom microbial interaction could be one of the key determinants in CD development [47]. Meanwhile, Liguori and collaborators showed that global fungus load increased in CD flare, predominantly by *Basidiomycota* and *Ascomycota* phyla. Meanwhile, *Filobasidium uniguttulatum* and *Saccharomyces cerevisiae* species were correlated with noninflamed mucosa, while *Xylariales* order was linked with inflamed mucosa [103]. A more recent study by Sokol et al. also revealed the distinctive mycobiome profile between IBD and healthy subjects. The authors reported a spike in Basidiomycota/Ascomycota ratio, accompanied with an expansion of *Malassezia sympodialis* and *C. albicans* and a decreased proportion of *Saccharomyces cerevisiae* in IBD in comparison with healthy controls. This study also highlighted the significant changes in fungal microbiota between remission and relapse stage within IBD cohort. For instance, *C. albicans* were remarkably increased during relapse when compared to remission stage. Lastly, based on the concomitant analysis of bacterial and fungal microbiota, the authors also suggest that an interkingdom interaction between fungi and bacteria could contribute towards IBD pathogenesis [9]. Hence, modulation of gut mycobiota could be a potential approach in the treatment of IBD [104].

#### 3.6.2. Inflammatory Bowel Syndromes (IBS)

Inflammatory bowel syndrome is a functional gastrointestinal disorder associated with altered bowel habits. Few studies have documented the link between IBS and fungal microbiota in the gut. Overall, the representation of fungi in healthy individuals is an insignificant proportion (~0.1%) of the entire microbiome as reported by various studies, with the three major fungal genera being *Saccharomycyes*, *Candida*, and *Cladosporium* [105]. An earlier study by Levine et al. concluded that overgrowth of *Candida* species is associated with diarrhea symptom in patients receiving antibacterial therapy [106]. Subsequently, another study documented that patients with antibiotic-associated diarrhea have *Candida* overgrowth in the gastrointestinal tract [107]. Furthermore, Santelmann and Howard reported that “IBS associated symptoms” or “Candida syndrome” could be triggered by *Candida* products, antigens and cross-antigens [108]. A more recent study by Botschuijver et al. demonstrated fungal dybiosis, predominant by *Saccharomyces cerevisiae* and *Candida albicans* in patients with IBS [51]. Taken together, the possible role of fungal microbiota in IBS remains much to explore. Additionally, whether a disruption in bacteria microbiota causes a skewed commensal mycobiota profile in IBS remains a question.

#### 3.6.3. Cancers

The gut mycobiome has been linked with the pathogenesis of colorectal adenoma, an inducer of colorectal cancer (CRC). A recent study by Luan et al. has discerned the role of gut fungi in different stages of colorectal adenoma. In the study, the authors characterized mucosal mycobiota in twenty-seven paired samples of adenomas and adjacent tissues, where *Glomeromycota* and *Basidiomycota* phyla dominated both adenomas and adjacent tissues from all subjects. In terms of genera, opportunistic pathogens *Phoma* and *Candida* were frequently identified (45%). Also, a decrease in fungal diversity in colorectal adenomas based on operational taxonomic unit (OTU) analysis was recorded [109]. Although the study illustrates the possible role of fungal microbiota in colorectal adenoma development, more studies are warranted to elucidate the cause-effect relationship between fungal microbiota and colorectal cancer. On the other hand, fungal dysbiosis is observed in patients with colorectal cancers and polyps. Additionally, the authors demonstrated that an increase in the Ascomycota/Basidiomycota ratio accompanied with the expansion of opportunistic fungi *Trichosporon* and *Malassezia* populations may facilitate the progression of colorectal cancer [110]. Similarly, Coker et al. reported fungal dysbiosis in patients with colorectal cancer. In addition to that, the authors also demonstrated an increase in the Basidiomycota : Ascomycota ratio with enrichment of fungal class *Malasseziomycetes* and depletion of classes *Pneumocystidomycetes* and *Saccharomycetes* were detected [111]. A recent study by Chin et al. has identified the presence of *Schizosaccharomyces pombe* in the guts of colorectal cancer patients and healthy subjects. The authors discovered that proteins secreted by *Schizosaccharomyces pombe* were present in high intensity in colorectal cancer patients, with four secreted proteins being closely related with the late stage of colorectal cancer [112]. Collectively, these studies suggest the undisputable role of fungi in colorectal cancer development and provide a new paradigm in utilizing these specific fungal signatures in disease diagnosis or therapeutic approaches.

#### 3.6.4. Hepatitis B and HIV Infections

Infectious viruses such as hepatitis B virus and HIV virus, which can affect host immunity, pose the potential to modify the role of other microbes in the gut including fungi, in the context of disease progression and exacerbation. One study characterized the association of fungi in the gut with varying degrees of chronic hepatitis B virus infection via culture-independent and culture-dependent analysis. In this study, Chen et al. reported high prevalence of *Aspergillus*, *Candida*, *Galactomyces*, *Saccharomyces*, and *Chaetomium* in patients with hepatitis B. Moreover, the authors surmised that richness and diversity of fungal species in hepatitis B patients are positively correlated with the disease progression in patients with chronic HBV infection [11]. Nevertheless, the positive correlation does not differentiate between cause and effect, which have yet to explored in recent studies.

On the other hand, considerable efforts investigating the relationship between gut mycobiota and HIV are also receiving much attention, as fungal infections often result in diarrhea wasting syndrome, as seen in HIV/AIDS [113]. Jha et al. assessed the clinical and microbiological profile of HIV/AIDS cases in association with diarrhea. The authors discovered that the prevalence of fungi was higher in HIV-seropositive patients in comparison with HIV-negative controls. The authors also discovered that *C. parvum*, *C. difficile*, and *C. albicans* are significantly present in HIV-seropositive patients [114]. Similarly, Awoyeni et al. reported an association between candidiasis and HIV patients with diarrhea. The most common species identified in this study were *C. albicans*, *C. krusei*, and *C. tropicalis* [115]. Meanwhile, Gouba and Drancourt also reported a reduction in fungal diversity in HIV-positive patients accompanied with higher prevalence of *Candida* species [116]. In another study, Esebelahie et al. reported that the prevalence of *Candida* in HIV patients without antiretroviral treatment was higher than HIV patients with active antiretroviral treatment. The *Candida* species recovered from the study were *C. albicans*, *C. glabrata*, *C. krusei*, *C. tropicalis*, and *C. parapsilosis* [117]. Collectively, these studies suggest that fungal microbiota especially *Candida* species are associated with the secondary immunodeficiency in HIV-positive patients, in the case of diarrhea and antibiotic treatment.

#### 3.6.5. Obesity

The association between fungi and obesity has been implicated in an obese subject with BMI of 48.9. In this study, an increase in fungal diversity is observed. Additionally, numerous fungi species identified in this study apparently originated from food sources (11 out of 16 fungal species). Nevertheless, the findings in this study may be overrepresented due to only one subject was involved in the study [118]. A recent study involving a higher number of obese subjects (*n* = 52) and controls was conducted. Based on the findings, there is a significant difference in the fungal composition between obese subjects and controls. Nonetheless, *Candida*, *Nakaseomyces*, and *Penicillium* were the most predominant genera identified in obese subjects, whereas *Mucor racemosus* and *M. fuscus* were the most represented in nonobese patients. Additionally, the authors found that *Mucor* genus was relatively increased in obese subjects upon weight loss. The authors surmise that targeting fungal communities in the gut could be a novel strategy in handling obesity [119].

#### 3.6.6. Diabetes

Micobiome analysis has been performed in a few studies to elucidate the role of microbiota in driving the pathogenesis of diabetes mellitus [120–122]. In the context of fungal involvement, Soyucen and collaborators found that besides *Echerichia coli*, *C. albicans* and *Enterobacteriaceae* colonization were increased in the patients with type 1 diabetes mellitus (T1DM), whereas *Bifidobacterium* colonization was reduced [123]. Apart from that, Gosiewski et al. measured the quantitative changes of *Candida s*pecies in both type 1 (T1DM) and type 2 diabetes mellitus (T2DM) patients via real-time PCR (qPCR). From the findings, *C. albicans* were predominant in the feces of patients with T1DM and T2DM. Nonetheless, there are no significant differences between T1DM and T2DM in terms of *C. albicans* colonization. Furthermore, the authors observed that the quantity of *Candida* is negatively correlated with serum lipids in T2DM patients [124]. Likewise, higher fungal species diversity was observed in T1DM with *C. albicans* being apparently less significant as compared to controls. The discrepancy could be due to greater recovery of fungal diversity in the T1DM cohort rather than a true reduction of *C. albicans* levels. Moreover, fungal species isolated from this study were resistant towards antifungal treatment [125]. Collectively, these studies showed that *Candida* species are more prevalent in patients with diabetes, particularly T1DM. This warrants further studies to elucidate the actual role of *Candida* in the pathogenesis of T1DM. Meanwhile, the role of gut fungi in T2DM remains much to be explored.

#### 3.6.7. Atherosclerosis

Recently, numerous studies have suggested the association of gut microbiota with the development of atherosclerosis and cardiovascular diseases [126–128]. Indeed, a recent preliminary study based on cardiovascular risk has documented the possible involvement of gut mycobiota in carotid atherosclerosis. This study recruits thirty-three obese subjects (men and women) where the risk of developing cardiovascular disease was determined through Framingham risk score (FRS) and the carotid intima-media thickness (cIMT). From the study, the authors identified the abundance of the phylum *Zygomycota*, which consists of family *Mucoraceae* and genus *Mucor* showing negative correlation with cIMT. Additionally, *M. racemosus* was the most prevalent species found in subjects with a low cardiovascular risk profile, and this species was negatively associated with FRS and cIMT. The authors suggest that *M. racemosus* could be a relevant biomarker for cardiovascular risk [129]. Thus, it seems that mycobiota could have its role in cardiovascular diseases and more studies are needed to unravel the beneficial effects of mycobiota in cardiometabolic diseases.

#### 3.6.8. Alcoholic Liver Disease

The role of gut mycobiota alcohol liver disease has been unraveled in a study involving animal model. In this study, Yang et al. observed an increased in fungal communities and *β*-glucan translocation into systemic circulation in mice after chronic alcohol administration. The authors reported that the effect of *β*-glucan-induced liver inflammation is mainly via C-type lectin-like receptor (CLEC7A) on Kupffer cells, which increases IL-1*β* expression, promotes hepatocyte damage, and consequently leads to the development of ethanol-induced liver disease. The authors further demonstrated a reduction in intestinal fungal growth and *β*-glucan translocation, followed by alleviation of ethanol-induced liver disease upon antifungal treatments. Meanwhile, the authors observed a decrease in fungal diversity along with *Candida* overgrowth in alcohol-dependent patients. Further, elevation in immune response against mycobiota was detected in alcoholic cirrhosis patients in comparison with patients with non–alcohol-related cirrhosis and healthy individuals. The authors surmised that chronic alcohol consumption is closely related with changes in gut mycobiota and translocation of fungal products. Thus, the authors suggest that manipulation of gut mycobiota could be an effective intervention for relieving alcohol-related liver disease [130]. A recent review by Szabo commented on the influence of fungal mycobiome on the complexity of alcohol-induced gut-liver axis from multiple aspects and some new insights to unravel the contribution of fungal microbiota between gut and liver in alcoholic liver disease [131].

#### 3.6.9. Neurological Disorders

On the other hand, gut mycobiota is considered as one of the etiological agents in driving the pathophysiology of central nervous diseases (both psychiatric and nonpsychiatric disorders). It is believed that exposure to infectious pathogens at the critical stages of neurodevelopment may deteriorate central nervous system and elicit behavioral anomalies and psychiatric disorders during adulthood [132, 133]. Strati et al. reported dysbiosis in both gut fungal and bacteria microbiota in subjects with Rett syndrome (RTT), a progressive neurological disorder frequently linked with constipation and gastrointestinal dysfunctions. The authors also reported that high abundance of *Candida* species was detected [134]. Meanwhile, in autism spectrum disorders (ASD), Strati et al. observed an alteration in both fungal and bacteria gut microbiota of ASD patients, with *Candida*, *Malassezia*, *Aspergillus*, and *Penicilliun* genera commonly identified. Further, the authors even observed an increase in *Candida* genus in autistic compared to neurotypical subjects in ASD [135]. Meanwhile, several studies suggested that schizophrenia, a psychiatric disorder, is associated with fungal dysbiosis, as portrayed by elevation of *S. cerevisiae* and *C. albicans* species [136]. Moreover, Severance et al. also demonstrated the presence of antibody against *C. albicans* and a robust link between gastrointestinal disturbances with elevated *C. albicans* in patients with schizophrenia and bipolar disorder, accompanied with lower cognitive scores in these patients [137]. Subsequently, the authors showed that supplementation with a probiotic *Lactobacillus rhamnosus* and *Bifidobacterium animalis* greatly improved psychiatric symptoms and normalized blood levels of *C. albicans* antibodies in schizophrenia [138]. A recent review by Forbes et al. also describes the recent advancement in knowledge regarding gut mycobiome and the plausible role of gut mycobiota in neurological disorders [139].

## 4. Challenges and Future Direction

Numerous sources have proven that gut mycobiota have certain weightage in maintaining host homeostasis. Nevertheless, there remain challenges that hindered the progression of fungal research and in-depth understanding on the fungal community in human. Culture-dependent methods utilizing conventional microbiological techniques, such as biochemical assays [140], microscopy [141], and observation of the fungal growth in culture media (Sabouraud dextrose agar and potato dextrose agar) [142], remain the preference for scientists in resolving the complexity of fungal community in microbial ecosystems. This is due to the fact that culture-dependent methods are inexpensive and cost effective in many laboratories, and alternatives to culture-dependent methods are yet to be developed.

On the other hand, advancement in the molecular era has shifted researchers towards nonculture-dependent methods in microbial community analysis. The approaches such as polymerase chain reaction (PCR) [143] and high-throughput next generation sequencing (NGS) technologies [144, 145] diversified fungal identification and analysis without the need of for complex culturomics [146, 147]. Nevertheless, gold standard methods for culture-independent analysis to study the complexity of gut mycobiota are still lacking. Recently published data using different molecular approaches such as denaturing gradient gel electrophoresis (DGGE) and cloning [148], qPCR and NGS [29], and NGS alone [149] in gut mycobiome studies raise the concern on the precision of these studies. There is no comparative analysis being undertaken to analyse and to corroborate the findings using nonculture-dependent methods thus far.

Furthermore, the number and significance of fungi in the gut may be underestimated due to several reasons. First, fungal analysis through sequencing efforts and matched with available annotated reference sequences may be underrepresented. Technical problems such as misspellings, poor annotation of fungal database, incomplete representation, and other factors may arise when assigning taxonomy to sequencing reads [150, 151]. Second, the gut fungal composition is influenced by a number of factors, including age, diet, host immunity, medication, host genetics, and also bacterial microbiome through interkingdom interactions [19, 30]. It remains largely unknown to what extent these factors might affect the diversity and stability of gut mycobiota throughout different developmental stages. Third, fungal cell is a substantial mass of biomaterial with its size approximately 100 times larger as compared to a typical bacterial cell, where simple genome-counting numbers may fail to characterize [7]. Lastly, there remain uncultivable fungi yet to discovered, with unknown function and taxonomy [7]. Hopefully, with the advance of culturomics study, the role of uncultivable fungi can be explored, and a full understanding of the fungal compartment in the gut can be attainable.

Although certain fungal species are commensals of human at various body parts, certain “foodborne fungi” or food contamination by fungi pose a serious threat to human health. “Foodborne fungi” illnesses can be caused by fungi from the genera *Aspergillu*s, *Alternaria*, *Fusarium*, *Candida*, and mucormycetes [152–154] or can be due to secondary metabolites such as mycotoxins. An estimation of 600 million foodborne diseases are reported annually [155], but the disease burden due to mycotoxin and fungus remains largely [156]. Moreover, we still lack a distinctive method to differentiate between foodborne fungi contaminants and commensal fungi that colonize the gut. The only distinguishable characteristic thus far is the visible clinical signs and symptoms often associated with foodborne microbial pathogens whereas the colonizers are often asymptomatic for the individuals that harbor the latter. A review mentioned that surrogate biomarkers such as procalcitonin in addition to the conventional but nonspecific neutrophil count could be used in conjunction with clinical evaluation for identifying infection as opposed to colonization [157]. Frequently, fungal contamination occurs during processing [158, 159], due to improper storage [160, 161], or due to the presence of fungi intrinsically in certain food products [162, 163]. Culturomics and noncultoromics methods are still actively being employed to identify fungi from both sources. Other possible future strategies could exploit the host factors to distinguish between the colonizers and the contaminants, since colonizers often elicit detectable immune responses whereas the colonizers do not cause untoward immune reactions or inflammations. Moreover, an ideal detection method should be developed to identify the presence of foodborne fungi at all stages of food production. As reviewed previously, a good detection tool should satisfy a few criteria such as high specificity and sensitivity, cost-effectiveness, is nonlaborious, and is not time consuming [164, 165].

Clearly, there is a strong connection between gut mycobiota and brain, and the potential of gut mycobiota in driving brain-associated diseases. Indeed, a review by Enaud et al. had proposed possible mechanisms involved in mycobiome-gut-brain axis interaction (GBA) [166]. Further, *Candida* species appear to be one of the most prevalent species identified in the studies discussed above. This indicates that *Candida* species could have notable role in the mycobime-GBA. Further studies should focus on the putative role of *Candida* species in this gut-brain interaction. Additionally, targeting gut mycobiota could be one of the potential intervention strategies to relieve neurological and neuropsychiatric disorders. For example, administration of *Saccharomyces boulardii* CNCM I-745 had improved intestinal neuromuscular anomalies in IBS-induced mouse model prior to *Herpes Simplex* Virus type 1 (HSV-1) exposure [167]. A recent study also documented the protective role of mycobiota in reducing inflammation in the central nervous system. In this study, Takata et al. reported that the administration of *C. kefyr* alleviated the severity of experimental autoimmune encephalomyelitis (EAE), an animal model of multiple sclerosis. The authors surmised that the protection could be due to alteration of bacterial microbiome, accompanied with increase of Tregs and regulatory dendritic cells in mesenteric lymph nodes and diminished production of T-helper 17 cells in the intestinal lamina propria [168].

## 5. Conclusion

From the accumulated evidences, it is clear that gut mycobiome has an indisputable role in host homeostasis and disease development, despite constituting only a small proportion in the gut. Nevertheless, the journey of uncovering the mystery of gut mycobiome must be continued. More multifaceted and multidisciplinary approaches have to be adopted in such a way to identify those uncultivatable or low abundance fungi in the gut, to characterize the fungal species and strain diversity in the gut, and also to differentiate permanent and transient fungal species that reside in the gut. In addition to that, interaction between fungi and their metabolites with various host players (brain, lungs, and host immune system) and xenobiotic components (diets, environments, and others) has to be emphasized, where this could offer new insight into the role of gut mycobiome on host physiology and disease development. Moreover, interkingdom interaction of various fungal species with other members (bacteria, parasite, and virus) present in the gut and how these interactions could affect us are areas to be further explored.

## Figures and Tables

**Table 1:** Significant involvement of fungi in human disease development.

Disease	Disease subtypes	Significant findings that involved fungi	Reference
Inflammatory bowel disease	Crohn's disease (CD)	(i) Fungal dysbiosis is closely related to CD in most of the conducted studies	[9, 47, 84, 101–103]
		(ii) Interkingdom interaction between fungal and bacteria was observed	[9, 47]

Inflammatory bowel syndrome (IBS)	—	(i) Fungal dybiosis, predominant by *Saccharomyces cerevisiae* and *Candida albicans* in IBS patients	[51]

Cancers	Colorectal cancer	(i) Fungal dysbiosis is observed in most of the reported studies	[109–112]

Infectious diseases	Hepatitis B	(i) High levels of *Aspergillus*, *Candida*, *Galactomyces*, *Saccharomyces*, and *Chaetomium* were identified	[11]
		(ii) Richness and diversity of fungal species is associated with chronic HBV infection	
	HIV	(i) *C. parvum*, *C. difficile*, and *C. albicans* are significantly present in HIV-seropositive patients	[114]
		(ii) *C. albicans*, *C. krusei*, and *C. tropicalis* were associated with diarrhea in HIV patients	[115]
		(iii) Fungal dysbiosis and high prevalence of *Candid*a species were observed in HIV patients	[116]
		(iv) Prevalence of *Candida* in HIV patients without antiretroviral treatment was higher than HIV patients with active antiretroviral treatment	[117]

Noncommunicable diseases	Obesity	(i) *Candida*, *Nakaseomyces*, and *Penicillium* genera were commonly identified in obese subjects	[119]
		(ii) *Mucor racemosus* and *M. fuscus* were identified in nonobese patients.	
		(iii) Specific fungal composition could be potentially used to distinguish between obese and nonobese patients	
	Diabetes	(i) *C. albicans* is more prevalence in type 1 diabetes	[123–125]
		(ii) *C. albicans* is more prevalence in type 1 and type 2 diabetes	
		(iii) No difference is found between *C. albicans* colonization in type 1 and type 2 diabetes.	[124]
		(iv) Isolated fungal species from type 1 diabetes patient is more resistant towards antifungal treatment	[125]

Atherosclerosis	—	(i) Phylum *Zygomycota*, which consists of family *Mucoraceae* and genus *Mucor*, was negatively correlated with the risk of cardiovascular disease development through carotid intima-media thickness (cIMT) method	[129]

Alcoholic liver disease	—	(i) Decreased in fungal diversity along with *Candida* overgrowth in alcohol-dependent patients	[130]

Central nervous system diseases	Rett syndrome	(i) High abundance of *Candida* genus were detected	[134]
	Autism Spectrum disorder	(i) *Candida*, *Malassezia*, *Aspergillus*, and *Penicilliun* genera were identified	[135]
	Schizophrenia	(i) Increased levels of *S. cerevisiae* and *C. albicans* species	[136]
		(ii) Close association of gastrointestinal tract disturbance with elevation of *C. albicans* species and lower cognitive score	[137]
